# Self-rated health status and associated factors in rural workers

**DOI:** 10.1186/s12889-023-15548-4

**Published:** 2023-04-12

**Authors:** Cleodice Alves Martins, Camila Bruneli do Prado, Júlia Rabelo Santos Ferreira, Eliana Zandonade, Olívia Maria de Paula Alves Bezerra, Luciane Bresciani Salaroli

**Affiliations:** 1 Graduate Program Nutrition and Health, Health Sciences Center, Federal University of Espírito Santo, Av. Marechal Campos, 1468, Maruípe, Vitória, ES 29040-090 Brazil; 2 Graduate Program Collective Health, Health Sciences Center, Federal University of Espírito Santo, Av. Marechal Campos, 1468, Maruípe, Vitória, ES 29040-090 Brazil; 3 Department of Family Medicine, Mental and Collective Health, Medical School, Federal University of Ouro Preto, Ouro Preto, Minas Gerais, Brazil

**Keywords:** Self-rated health, Rural workers, Health status indicators

## Abstract

**Background:**

Self-rated health status can be considered a good predictor of morbidity and mortality and has been used due to its easy assessment and applicability. The instrument is efficient for understanding sociodemographic, environmental and clinical conditions that may be related to the self-rated health status. Thus, this study aims to analyze the self-assessment of health status in rural workers and its association with socioeconomic characteristics, lifestyle, clinical condition and work characteristics.

**Methods:**

This is a cross-sectional study carried out with 787 male and female rural reporting agriculture as their main source of income in the municipality of Santa Maria de Jetibá. A simple and direct question was used “In general, compared to people your age, how do you rate your own state of health?” to see how rural workers rate their current health status. The independent variables analyzed were socioeconomic, clinical, health and work conditions. The magnitude of the associations was evaluated by means of hierarchical logistic regression.

**Results:**

It was found that 42.1% of rural workers self-rated their health status as regular or poor. Belonging to socioeconomic classes C (OR = 1.937; 95% CI = 1.009–3.720) or D/E (OR = 2.280; 95% CI = 1.178–4.415), being overweight (or having excess weight) (OR = 1.477; 95% CI = 1.086–2.008), multimorbidity (OR = 1.715; 95% CI = 1.201–2.447) and complex multimorbidity (OR = 1.738; 95% CI = 1.097–2.751) were risk factors for worse self-rated health.

**Conclusion:**

It was concluded that chronic diseases, socioeconomic status and overweight are risk factors for negative self-rated health. The identification of these determinants through self-rated status can support the planning of actions aimed at improving the health of the rural population.

**Trial registration:**

This study was approved by the Research Ethics Committee of the Health Sciences Center of the Federal University of Espírito Santo (Protocol No. 2091172; CAAE No. 52839116.3.0000.5060). All research participants gave their informed consent.

## Background

The health of workers is conditioned by socioeconomic, lifestyle, medical condition and occupational factors [1]. The distinction between the characteristics of rural work and other activities is very particular, and among such differences we highlight the exhaustive working day, worker exposure to different weather conditions, contact with potentially harmful animals and plants, indiscriminate use of agricultural products, poor hygiene conditions, difficult access to health and education services, and low remuneration [2, 3].

A considerable portion of rural workers, especially those who handle pesticides without the correct use of individual protective equipment (IPE), can suffer impacts on their quality of life and consequently increased morbidity and mortality, which directly affect rural work [4–6]. Added to this is the difficult access to health services, especially in primary care [7], and as a result there is a combination of risk factors inherent to working in the field that demand special attention for this population.

It is noteworthy that the share of the agricultural sector in Brazil's gross domestic product (GDP) is 27.4%, the highest rate in the last 20 years [8]. However, the success of economic indicators does not reflect on social indicators and reflects even less on the working conditions and health of rural workers [1].

Self-rated health, a construct that involves physical, mental and social aspects of life through the individual's general perception of his/her health [9], has been widely used in epidemiological studies because it is a simple, subjective, easy-to-evaluate and applicable measure [10, 11]. It is also used for comparing health services and resource needs according to geographic areas and to calculate morbidity and mortality indicators [12–14] and functional decline [15].

In rural areas, the evidence on morbidity and actual health conditions is limited and inconsistent [16, 17]. Use of the self-rated health indicator can be a powerful tool to elucidate the determinants and conditions of health manifested in the rural environment and to support the planning of health care for this population. Thus, this study aims to analyze the self-rated health status in rural workers and its association with socioeconomic characteristics, lifestyle, clinical condition and work characteristics.

## Methods

### Study design, setting and participants

This is a cross-sectional study derived from a larger study entitled “Health condition and associated factors: A study of rural workers in Espírito Santo—AgroSaúdES”, funded by the Espírito Santo Research Support Foundation (FAPES; Grant FAPES/CNPq/Decit-SCTIE-MS/SESA-PPSUS No. 05/2015).

The study was carried out in the city of Santa Maria de Jetibá,located in the state of Espírito Santo, southeastern Brazil, and had the participation of male and female rural workers registered in the Family Health Strategy (FHS) whose main source of income was agriculture. More information about data collection and other research details can be found in a previous study [18].

The inclusion criteria considered were: age 18–59 years, not being pregnant, having agriculture as the main source of income and being in full employment for at least six months.

### Data collection

Data collection took place between December 2016 and April 2017 on the premises of the health units of the studied municipality. A semi-structured questionnaire was administered with questions about socioeconomic, demographic and occupational characteristics, occupational contact with pesticides, lifestyle, eating habits, health status and self-rated health. In addition, anthropometric data were collected.

### Measurements

Self-rated health status was assessed through the question: “In general, compared to people your age, how do you rate your own state of health?”. Possible answers were “very good”, “good”, “regular” and “bad”. For analysis, the variable was recategorized as “good/very good” and “fair/poor”.

Independent variables related to socioeconomic characteristics, lifestyle, health and work conditions were self-reported by the research participants. Sociodemographic variables included gender, age group, schooling, ethnicity, marital status, economic class and ties to the land.

Socioeconomic class was defined according to the Brazilian Economic Classification Criteria [19], where A and B are considered the highest economic levels, C as the intermediate level and D and E as low economic levels. Age group was categorized as “up to 29 years old”, “30–39 years old”, “40–49 years old” and “over 50 years old”, while schooling was established according to the number of years of study reported by the participant.

In relation to lifestyle, rural workers who reported smoking or not smoking but had smoked in the past were considered as a “current/previous smokers” and those who reported never having smoked as “non-smokers”. Alcohol intake was assessed by asking "How often do you drink alcohol?" and categorized as “never”, “less than once a month” and “more than once a month”. The rural workers were also asked if they performed any other physical activity besides those related to agricultural work. Individuals who accumulated at least 30 min of physical activity per day, at least 5 days a week, of moderate intensity were classified as physically active and the others were classified as physically inactive [20].

Body mass index (BMI) was defined from weight (kg) and height (m) data using the formula: BMI = Weight/(Height)^2^. World Health Organization (WHO) cut-off points were used for the classification of individuals as low weight (BMI < 18), normal (≥ 18.5 to < 25), overweight (≥ 25 to < 30) or obese (≥ 30) [21]. Subsequently, for a better analysis, the data were categorized into eutrophic/underweight and overweight/obesity. Waist circumference was also classified according to the WHO, considering values of ≤ 94 cm for men and ≤ 80 cm for women as “adequate” and values above these as “high/very high” [21].

To assess multimorbidity the presence of two or more chronic diseases was considered [22] and for complex multimorbidity the occurrence of three or more chronic conditions that affect three or more different body systems or domains was considered [23]. To determine the affected systems according to each disease, we used the 11^th^ revision of the International Classification of Diseases (ICD-11): circulatory system (hypertension, stroke, heart attack, cardiac arrhythmia); endocrine, nutritional or metabolic disorders (diabetes, dyslipidemia, thyroid disorders); musculoskeletal or connective tissue system (arrhythmia); mental, behavioral or neurodevelopmental disorders (Alzheimer's, depression); genitourinary system (infertility, kidney disease); digestive system (liver cirrhosis); pulmonary system (bronchitis, asthma, pulmonary emphysema); and neoplasms (cancer) [18].

With regard to occupational characteristics, the following were evaluated: workload in hours per working week (time as a rural worker was categorized as “less than 10 years”, “10–29 years” and “over 30 years”); type of production; number of crops worked; contact and frequency of contact with pesticides, in addition to the amount of pesticides frequently in contact with; time of exposure to pesticides; and the use of IPE.

### Statistical methods

Absolute and relative frequencies of the independent variables were calculated, according to the categories of the rural workers’ self-assessment of health status. A chi-square test was performed to verify the association between the independent variables and the outcome. Variables with *p* < 0.20 were included in the multivariate analysis.

Binary logistic regression was performed with five models according to previous studies [24, 25]. For the first four models, the Enter method was used to collect socioeconomic, behavioral, anthropometric, work and health variables, respectively. For the fifth model the Forward method was used to enter the predictor variables one by one according to their contribution to the model, leaving only statistically significant variables at the end. Adjusted odds ratios (OR), 95% confidence intervals (CI) and a significance level of 5% were presented.

### Ethical aspects

This study followed all the ethical precepts of the Declaration of Helsinki and was approved by the Research Ethics Committee of the Health Sciences Center of the Federal University of Espírito Santo (Protocol No. 2091172; CAAE no. 52839116.3.0000.5060). All research participants gave their informed consent for study participation.

## Results

Of the total 787 rural workers eligible for this study, 57.9% (*N =* 456) self-rated their health status as good or very good and 42.1% (*N =* 331) as fair or poor. Gender (*p* = 0.01) and socioeconomic class (*p* = 0.004) showed a statistical difference between the proportions of the categories (Table 1).

**Table 1:** Self-rated health status according to socioeconomic characteristics of rural workers

	Self-rated health status				*p*-value*
	Very good/good		Regular/poor		
	N	%	N	%	
Gender					0.001
Male	261	57.2	150	45.3	
Female	195	42.8	181	54.7	
Age group					0.113
≤ 29 years	131	28.7	82	24.8	
30–39 years	140	30.7	90	27.2	
40–49 years	98	21.5	95	28.7	
50 years or more	87	19.1	64	19.3	
Education					0.096
< 4 years	294	64.5	237	71.6	
4–8 years	107	23.5	65	19.6	
8 years or more	55	12.1	29	8.8	
Ethnicity					0.168
White	399	87.5	300	90.6	
Non–white	57	12.5	31	9.4	
Marital Status					0.126
Unmarried	41	9.0	18	5.4	
Married or living with partner	382	83.8	293	88.5	
Separated, divorced or widowed	33	7.2	20	6.0	
Socioeconomic class					0.004
Class A or B	44	9.6	14	4.2	
Class C	234	51.4	160	48.3	
Class D or E	178	39.0	157	47.4	
Ties to the land					0.210
Owner	359	78.7	248	74.9	
Non–owner	97	21.3	83	25.1	

^*^Chi-square Test

Table 2 shows the proportional differences in behavioral, anthropometric and health condition variables according to the self-assessment of health status. There is a statistical difference in BMI, waist circumference, multimorbidity and complex multimorbidity (overall *p* = <0.001).

**Table 2:** Self-rated health status according to the behavioral and anthropometric characteristics of rural workers

	Self-rated health status				*p*-value*
	Very good/good		Regular/poor		
	N	%	N	%	
Alcohol intake					0.366
Never	249	54.6	191	57.7	
Less than once a month	113	24.8	85	25.7	
More than once a month	94	20.6	55	16.6	
Smoking					0.756
No smoking	382	83.8	280	84.6	
Current/past smoking	74	16.2	51	15.4	
Physical activity off field					0.251
No practice of physical activity	370	81.1	274	82.8	
Below recommended	46	10.1	38	11.5	
Within recommended	40	8.8	19	5.7	
Body mass index					< 0.001
Low weight/normal	253	55.5	133	40.2	
Overweight/obese	203	44.5	198	59.8	
Waist circumference					< 0.001
Adequate	249	54.7	131	39.6	
High/Very high	206	45.3	200	60.4	
Multimorbitidy					< 0.001
No	309	67.8	151	45.6	
Yes	147	32.2	180	54.4	
Complex multimorbidity					< 0.001
No	408	89.5	247	74.6	
Yes	48	10.5	84	25.4	

^*^Chi-square Test

The bivariate analysis between self-assessment of health status and work characteristics is described in Table 3. It is observed that, in this case, only the time working as a farmer showed a statistical difference (*p* = 0.047).

**Table 3:** Self-rated health status according to the work characteristics of rural workers

	Self-rated health status				*p*-value*
	Very good/good		Regular/poor		
	N	%	N	%	
Workload (hours/week)					0.220
≤ 40	87	19.1	75	22.7	
> 40	369	80.9	256	77.3	
Time as a farmer					0.047
< 10 years	28	6.2	9	2.7	
10–29 years	233	51.3	164	49.5	
30 years or more	193	42.5	158	47.7	
Type of production					
Conventional	405	88.8	303	91.5	0.209
Not conventional	51	11.2	28	8.5	
Number of crops worked					
≤ 4 crops	208	45.6	137	41.4	0.238
5 crops or more	248	54.4	194	58.6	
Contact with pesticides					
Direct	316	69.3	231	69.8	0.883
Indirect, organic or agroecological	140	30.7	100	30.2	
Frequency of contact with pesticides					
Daily/weekly	250	59.2	200	63.9	0.296
Monthly/annual	121	28.7	85	27.2	
No contact	51	12.1	28	8.9	
Number of pesticides usually in contact with					
None	140	32.6	100	31.4	0.775
≤ 5 types	130	30.3	92	29	
> 5 types	159	37.1	126	39.6	
Years of exposure to pesticides					
≤ 20 years	150	48.9	112	48.9	0.991
20 years or more	157	51.1	117	51.1	
Use of IPE^a^					
No or incomplete use	216	48.3	162	50.3	0.803
Complete use	91	20.4	60	18.6	
Direct/organic/agroecological contact	140	31.3	100	31.1	

^*^ Chi-square Test

^a^ *IPE* Individual protection equipment

Table 4 shows the logistic regression models for the variables that showed *p* < 0.20 in the bivariate analysis. Gender, socioeconomic class, BMI, multimorbidity and complex multimorbidity were associated with the self-rated health. It was observed that males showed a reduction of 30% (*p* = 0.022; OR = 0.705; 95% CI = 0.522–0.951) in the chance of individuals negatively self-evaluating their own health. In addition, being in socioeconomic classes C (*p* = 0.047; OR = 1.937; 95% CI = 1.009–3.720) and D/E (*p* = 0.014; OR = 2.280; 95% CI = 1.178–4.415) almost doubled the chance of individuals negatively self-evaluating their own health. Being overweight was also shown to be a risk for negative self-rated health (*p* = 0.013; OR = 1.477; 95% CI = 1.086–2.008). Finally, the conditions of multimorbidity (*p* = 0.003; OR = 1.715; 95% CI = 1.201–2.447) and complex multimorbidity (*p* = 0.018; OR=1.738; 95% CI = 1.097–2.751) almost doubled the chance of individuals perceiving their health status as fair or poor. It is interesting to note that the same variables showed statistical significance independent of the model, as they were added to the analysis. This means that, in the case of the self-rated health status of rural workers, the socioeconomic, anthropometric, work and health condition variables did not influence each other but acted as a set of risk factors for the health of this population.

**Table 4:** Association between self-assessment of health status socioeconomic and anthropometric factors and the work characteristics of rural workers (*N*=787)

Categoria	Model 1	Model 2	Model 3	Model 4	Model 5
	OR (95 CI%)	OR (95 CI%)	OR (95 CI%)	OR (95 CI%)	OR (95 CI%)
**Gender**					
Female	1	1	1	1	1
Male	0.674* (0.501–0.905)	0.728 (0.524–1.011)	0.715* (0.513–0.996)	0.709* (0.505–0.994)	0.705* (0.522 – 0.951)
**Ethnicity**					
White	1	1	1	1	
Non-white	0.745 (0.465–1.196)	0.769 (0.477–1.241)	0.772 (0.479–1.246)	0.721 (0.441–1.177)	
**Education**					
< 4 years	1.341 (0.812–2.216)	1.176 (0.707–1.958)	1.087 (0.640–1.846)	1.130 (0.657–1.943)	
4–8 years	1.074 (0.613–1.882)	1.019 (0.579–1.793)	0.994 (0.562–1.757)	0.996 (0.557–1.781)	
8 years or more	1	1	1	1	
**Marital Status**					
Unmarried	1	1	1	1	
Married or living with partner	1.374 (0.755–2.501)	1.311 (0.715–2.403)	1.217 (0.655–2.260)	1.251 (0.666–2.350)	
Separated, divorced or widowed	0.939 (0.415–2.128)	0.889 (0.392–2.062)	0.823 (0.351–1.927)	0.800 (0.335–1.911)	
**Socioeconomic class**					
Class A or B	1	1	1	1	1
Class C	1.861 (0.974–3.553)	1.971* (1.027–3.783)	2.003* (1.042–3.852)	1.806 (0.931–3.503)	1.937* (1.009–3.720)
Class D or E	2.294* (1.186–4.438)	2.480* (1.274–4.826)	2.486* (1.276–4.843)	2.166* (1.100–4.265)	2.280* (1.178–4.415)
**Body mass index**					
Low weight/normal		1	1	1	1
Overweight/obese		1.629* (1.081–2.455)	1.633 (1.084–2.462)	1.445 (0.948–2.203)	1.477* (1.086–2.008)
**Waist circumference**					
Adequate		1	1	1	
High/Very high		1.138 (0.733–1.777)	1.106 (0.707–1.731)	0.994 (0.628–1.572)	
**Time as a farmer**					
< 10 years			1	1	
10–29 years			1.684 (0.733–3.862)	1.532 (0.662–3.547)	
30 years or more			1.731 (0.718–4.170)	1.300 (0.531–3.183)	
**Multimorbitidy**					
No				1	1
Yes				1.753* (1.215–2.530)	1.715* (1.201–2.447)
**Complex multimorbidity**					
No				1	1
Yes				1.814* (1.140–2.885)	1.738* (1.097–2.751)

^*^ *P*-values < 0.05

*CI* Confidence interval, *OR* Odds ratio

Model 1: socioeconomic variables; Model 2: socioeconomic, behavioral and anthropometric variables; Model 3: socioeconomic, behavioral, anthropometrics and work characteristics variables; Model 4: socioeconomic, behavioral, anthropometric, work characteristics and health condition variables; Model 5: final model using the Forward method of logistic regression for the selection of variables

## Discussion

This study showed a high prevalence of rural workers who self-rated their health as fair or poor, which was mainly associated with socioeconomic class, BMI and multimorbidity conditions, given that self-rated health status is an easily applicable indicator that considers biological, psychological, social, demographic and cultural factors, along with factors related to the living and working environment [24]. It is extremely important to understand how these factors affect rural populations so that the proper measures can be taken to prevent and protect the health of these individuals. The scarcity of studies on the health of rural populations in Brazil, the unique aspects associated with this population and the close relationship between self-rated health and morbidity/mortality [26] highlight the relevance and urgency of studying this topic for improving public health.

Self-assessment of health is widely used in Brazilian and international epidemiological studies because it has a strong predictive power for morbidity and mortality and the use of health services, in addition to its overall assessment of symptoms, illness and an individual's general well-being [27–31].

In the present study, the number of individuals who self-rated their health as fair or poor was greater than that observed in similar studies involving rural workers [17] and agricultural areas [16]. Recent population data from the Surveillance of Risk and Protective Factors for Chronic Diseases by Telephone Survey (VIGITEL) pointed out that 4.7% of the individuals evaluated their health status negatively, with this proportion being higher in women (5.5%) than in men (3.7%). In the capital of the state of Espírito Santo, this percentage was even lower, with a negative self-assessment of health by only 3.5% of the population [32]. Despite the methodological differences between VIGITEL and the present study, which limit comparison, the discrepancy between the prevalences found demonstrates the urgency of practical actions aimed at the health of individuals living in rural areas, where the prevalence of negative self-rated health is much higher and access to health services is difficult.

In accordance with what is established in the scientific literature [17, 33, 34], the present study found that women have a greater negative perception of their health status. One of the justifications for this fact may lie in the feeling of denial of weakness and rejection of help regarding health-care among men [35]. In this context, it may be that women are more attentive than men when faced with health problems and are more attentive to minor problems in their subjective assessment of health [36].

In the rural setting, it is worth noting that women have both limited access and accessibility to health services. Although used as synonyms, these terms have complementary meanings. Access concerns the provision of health services, which allows timely use to achieve the best possible results, that is, the way in which the person experiences the available service. Accessibility means the possibility that people have or do not have access to services [37]. The rural environment still has strong gender constructions that value male hegemony and female submission in decisions and opportunities, preventing the socio-institutional support [38].

The sociodemographic conditions should also be highlighted in the rural context. In the present study, there was a risk gradient for negative self-assessment of health status as socioeconomic class decreased. This finding has already been well established in the scientific literature, indicating that a higher socioeconomic level is associated with better self-rated health [24, 39, 40].

Favorable socioeconomic conditions directly influence a good assessment of health status [41]. This relationship can be explained by the greater purchasing power of materials and structural conditions in the higher classes, which have the potential to shape psychosocial factors and health behavior, favorably influencing the perception of health [39, 42].

In the specific case of rural workers, it is important to draw a historical parallel and mention the strong changes that took place after the Green Revolution, which aimed to maximize crop yields in different ecological situations [43] through genetic improvements in plants and the evolution of production apparatus [44, 45]. Such changes, marked by the mechanization of rural work, directly affected family-based agriculture, increasing inequality in the distribution of land and causing family agriculture to occupy a secondary and subordinate place in society, marked by struggles to gain space. itself in the economy [46–48].

Currently, according to data from the Food and Agriculture Organization of the United Nations [49], about 80% of world food production comes from family farming, occupying 18% of cultivated land in South America. In Brazil, more than 80% of agricultural exports are of the family type, which is why the country stands out as the eighth largest food producer in the world in this segment [50]. However, studies indicate that the gross monthly production value per family property is around 0.46 minimum wage, which represents a large part of the producers in extreme poverty. This reality reflects a risk to the sustainability of family farms [51] and partly explains the vulnerability that rural workers find themselves in when it comes to health.

In the present study, an association was found between excess weight, assessed through the BMI, and a negative self-assessment of health status, consistent with the scientific literature [52, 53]. This relationship has also been shown to be mediated by obesity-related comorbidities [54], such as cardiovascular disease and cancer [55, 56]. Furthermore, obesity is associated with reduced physical activity and lower exercise capacity, both conditions associated with low self-rated health [54].

In a longitudinal study, high BMI was associated with negative self-rated health, regardless of comorbidities. However, during the years of follow-up of the study, individuals who lost weight changed their perception of health previously evaluated as poor [52], showing that the BMI is an important predictor of how the individual evaluates his/her own health.

Conceptually, obesity is the result of several factors, and in this context the "environment" stands out, capable of directly affecting the individual's eating behavior and physical activity, and consequently, the energy balance [57]. For the authors, one of the environments is the "perceived environment", which highlights the individual's perception of the spaces in which he is inserted, such as the distance to leisure and food facilities [58] and consequently also to health services.

Agriculture is often described as a healthy occupation, associated with an image of a favorable lifestyle, with exposure to nature, outdoors, physical exertion and a diet based on natural foods [59]. However, this has not been the reality found in agricultural work. In addition to work-related injuries, such as physical trauma/injury and respiratory diseases, an increase in the prevalence of chronic diseases, such as arterial hypertension, dyslipidemia, diabetes and metabolic syndrome, is observed in the countryside [60–65]. Furthermore, morbidity and mortality rates from chronic health conditions are higher among rural populations compared to urban populations [66].

In this scenario enter the concepts of multimorbidity and complex multimorbidity. According to the WHO [67], multimorbidity is defined by the presence of two or more chronic diseases in the same individual. Complex multimorbidity is defined as the occurrence of three or more chronic conditions that affect three or more different body systems or domains [23].

In this study, individuals with multimorbidity and complex multimorbidity were more likely to perceive their health status as fair or poor. Petarli et al. [5], analyzing the same group of rural workers, showed the prevalence of multimorbidity and complex multimorbidity to be 41.5% and 16.7%, respectively. The most prevalent conditions were arterial hypertension, dyslipidemia and depression. Corroborating our findings, a controlled study in a rural population in China found that the presence of chronic diseases impacted changes in self-rated health status scores [67].

Agricultural work, added to sociodemographic characteristics and the reduced availability of health services, makes rural workers vulnerable to the implications and consequences of multimorbidity. The occurrence of multiple chronic conditions increases the demand for more complex care, creating a paradox between the need and the difficulty of reaching health services. This situation causes direct impacts for the patient, professionals and the health system, among which we can mention: complex health treatments, with potentially competing priorities and therapeutic goals [68]; a higher number of outpatient consultations and hospital admissions [69]; polypharmacy [70] and the higher cost of medicines and treatments [71]; and negative labor impacts, such as lower productivity and higher risk of unemployment [72].

Finally, considering that about 15 million people are currently engaged in agricultural activities in Brazil, the implications of reduced productivity associated with multimorbidity would be quite serious. Not only would the farmer be financially harmed, but this situation could compromise the food security of the population that consumes the food produced, and more broadly the country's GDP, since a large portion is dependent on agricultural activity [71].

The results of this study must be interpreted within the context of its limitations. Among them, the methodological design stands out, which does not allow for causal or temporal inferences about the associations found. In addition, some measures were based on self-report and therefore may be subject to recall bias, diagnostic suspicion and socially desirable responses. However, it is noteworthy that this is a population-based study with the assessment of aspects related to health having an unprecedented character in relation to the target population involved.

## Conclusion

Gender, socioeconomic class, BMI and the conditions of multimorbidity and complex multimorbidity of rural workers were associated with the self-perception of health status. The results show that it is necessary to reassess the access and focus of the health system in rural areas, in addition to strengthening general primary care, with an appropriately qualified multidisciplinary team, so that holistic and continuous care is promoted in these populations.

## Data Availability

Dataset used and/or analyzed during the current study are available from the corresponding author upon reasonable request.
